# Effect of Ga_2_O_3_ Dopant on High Speed Sintered 5 mol% Y_2_O_3_ Stabilized Dental Zirconia

**DOI:** 10.3390/ma16020714

**Published:** 2023-01-11

**Authors:** Kazumichi Nonaka, Mitsuji Teramae, Giuseppe Pezzotti

**Affiliations:** 1 Department of Research and Development, SHOFU INC., Higashiyama-ku, Kyoto 605-0983, Japan; 2 Ceramic Physics Laboratory, Kyoto Institute of Technology, Matsugasaki, Sakyo-ku, Kyoto 606-8585, Japan

**Keywords:** zirconia, dental material, gallium doping, high-speed sintering

## Abstract

The high-speed sintering of zirconia has become essential for ceramic dental prosthesis treatment in a single visit. Previous studies have shown that 5 mol% yttria-stabilized zirconia (5Y zirconia), with the exception of some types, loses strength and translucency with high-speed sintering. In this study, 0.15–0.92 wt% Ga_2_O_3_, which is expected to promote the sintering of zirconia, was added to improve the properties of 5Y zirconia high-speed sintered bodies, and the effect of its addition was evaluated. The specimens were characterized by density and translucency measurements, a three-point bending test, X-ray diffraction (XRD), scanning electron microscopy (SEM), and shrinkage measurement. The addition of Ga_2_O_3_ improved both translucency and flexural strength of 5Y zirconia high-speed sintered bodies. XRD and SEM observations revealed that this improvement in properties was due to the change in the crystal phase composition and the decrease in the amount and size of pores due to the addition of Ga_2_O_3_. Shrinkage measurements also revealed that the addition of Ga_2_O_3_ changed the sintering behavior of 5Y zirconia, suggesting that this change led to a reduction in porosity. From the above results, it was concluded that Ga_2_O_3_ addition is effective in improving the properties of 5Y zirconia high-speed sintered bodies.

## 1. Introduction

Zirconia has become a major dental prosthetic material due to its excellent mechanical properties, chemical stability, high aesthetics, radiopacity and biocompatibility [1,2]. Zirconia sintered bodies undergo a phase transformation from a metastable tetragonal phase to a monoclinic phase accompanied by volume expansion when external stress is applied. This is known as a stress-induced phase transformation and is responsible for the excellent mechanical properties of zirconia sintered bodies [3]. Since pure zirconia is only stable as a monoclinic phase at room temperature, oxides called stabilizers (CaO, MgO, CeO_2_, Y_2_O_3_) are often added to zirconia to stabilize the tetragonal phase and allow it to exist at room temperature. Of these stabilizers, Y_2_O_3_ is most commonly used for dental zirconia. The concentration of the stabilizer is an important factor for dental zirconia because the properties of zirconia are greatly affected by the concentration of the stabilizer: the mechanical properties improve and translucency decreases with increasing stabilizer concentrations [4,5]. In the field of prosthetic dentistry, 3 mol% Y_2_O_3_ stabilized zirconia (3Y zirconia), 4 mol% Y_2_O_3_ stabilized zirconia (4Y zirconia), and 5 mol% Y_2_O_3_ stabilized zirconia (5Y zirconia) are mainly used. These materials are selected according to the clinical cases. For example, 5Y zirconia is used in cases with high esthetic demands such as anterior teeth due to its high translucency, and 3Y zirconia is used in the presence of heavy loads such as the case of long span bridges [6,7].

Zirconia dental prostheses are made with a computer-aided design and manufacturing (CAD/CAM) approach. Since zirconia sintered bodies are too hard and strong to be machined, semi-sintered zirconia is first processed into a desired shape and then further sintered to full density. Since this sintering usually takes 6 to 10 h [8], the production efficiency of zirconia prostheses is not high. Therefore, improvements are required to shorten the sintering time.

In recent years, several sintering furnaces capable of high speed sintering have been released. Among them, the most distinctive example is CEREC Speedfire (Dentsply Sirona). Zirconia can be densified in 30 min with employing this sintering furnace, so dentists can complete zirconia prosthetic treatment in one day (One Visit Treatment) [9,10]. Generally, zirconia prostheses using this system often use monolithic zirconia without porcelain build-up because of technical and time constraints. A zirconia prosthesis is typically made by a dental technician. In cases requiring high esthetic appearance, dental technicians build multiple layers of porcelain onto the zirconia coping to create a prosthesis that mimics the appearance of natural teeth. This process requires skilled techniques and long hours of work. However, with the one visit treatment, it is necessary to fabricate a zirconia prosthesis in a short period of time. Therefore, porcelain build-up is abandoned in many cases. In order to fabricate a monolithic zirconia prosthesis with high aesthetics, it is necessary to use a zirconia sintered body with high translucency (e.g., 5Y zirconia).

It is well known that the various properties of ceramic sintered bodies are affected by sintering conditions such as heating rate and holding time. A number of reports have been published on the various properties of high-speed sintered dental zirconia [11,12,13,14,15,16]. Despite some differences in those reports on the effect of high-speed sintering on various properties of zirconia sintered bodies, it is generally accepted that a key-factor in translucency resides in the choice of yttria concentration. In 3Y and 4Y zirconia, high-speed sintering does not significantly reduce mechanical properties and translucency, but in 5Y zirconia, depending on the type of 5Y zirconia, pores remain in the sintered body, which significantly decrease both the translucency and the mechanical properties [13,15]. Therefore, high-speed sintering is not applicable for some 5Y zirconia.

The formation of residual pores in zirconia sintered bodies is affected by their sintering behavior. The addition of metal oxides is known as a means of changing the sintering behavior of ceramics. The effect of oxide additives on the sintering behavior of zirconia was reported in a report by Flegler et al. [17]. Their study showed that the addition of alkaline earth metals inhibited sintering, while the addition of most transition metals accelerated it. In addition, a small amount of alumina is added as a sintering aid to most dental zirconia [5,11]. Matsui et al. [18] first reported the effects of small amounts of alumina addition to the sintering behavior of yttria-stabilized zirconia. It was clarified that the addition of alumina promotes sintering by changing the sintering mechanism from grain boundary diffusion to volume diffusion. Yoshida et al. [19] reported that small cations segregated at the grain boundaries promote grain boundary diffusion. Based on these reports, it is possible that the addition of oxides of small trivalent metals promotes the sintering of zirconia. Therefore, we focused on Ga_2_O_3_. Ga^3+^ (0.62 Å, 6-fold) is a trivalent cation that is smaller than Zr^4+^ (0.72 Å, 6-fold). Furthermore, the colorlessness of Ga_2_O_3_ is a favorable factor because it does not affect the color tone of zirconia. Since Ga is not a dominant metal in dentistry, the biological effects of using Ga_2_O_3_ in dental materials are unknown. However, Cochis et al. [20] reported that Ga alloys were not cytotoxic, suggesting that the more stable oxide Ga_2_O_3_ is also not cytotoxic.

Although the effect of adding a number of different metal oxides has been investigated, to the best of our knowledge, there are no reports with regard to the adding of Ga_2_O_3_ to yttria-stabilized zirconia. The present study aims to investigate the effect of the addition of Ga_2_O_3_ on the properties of high-speed sintered 5Y zirconia and to clarify the causes from the changes in crystal structure, microstructure, and sintering behavior.

## 2. Materials and Methods

### 2.1. Preparation of Test Piece

**Figure 1:**
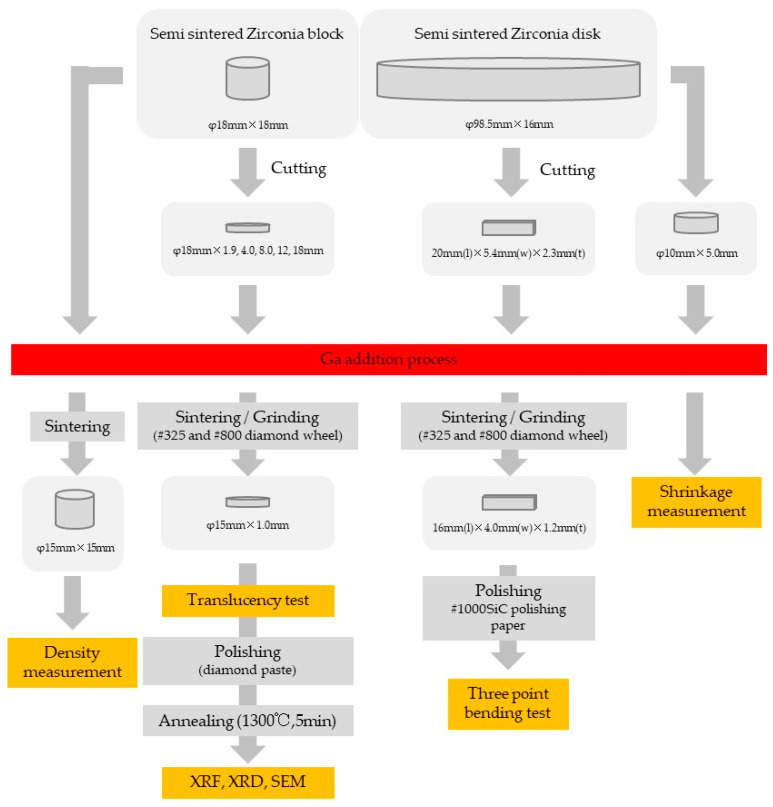
Preparation procedure of the specimen and specimen dimensions (The mark “φ” denotes “diameter”).

Figure 1 and Table 1 and Table 2 show the dimensions of the specimen and the fabrication procedure.

**Table 1:** Raw material powder and Ga solution used.

Sample Name	Raw Material Powder	Chemical Composition of Zpex Smile (wt%) *	Ga Solution **
Ga0	Zpex Smile	ZrO_2_: 88.84 Y_2_O_3_: 9.29 Al_2_O_3_: 0.049 HfO_2_: 1.82	-
Ga1.5			GS1.5
Ga2.5			GS2.5
Ga5.0			GS5.0

* Manufacturer analysis value. ** Table 3.

**Table 2:** Specimen dimensions in each test.

Measurement	Pre-Sintered Sample Size	Measured Sample Size
Density	φ18 mm × 18 mm	φ15 mm × 15 mm
XRD	φ18 mm × 1.9 mm	φ15 mm × 1.0 mm
SEM	φ18 mm × 1.9 and 18 mm	φ15 mm× 1.0 mm
Translucency	φ18 mm × 1.9, 4.0, 8.0, 12 and 18 mm	φ15 mm × 1.0 mm
Three-point flexural test	20 mm (l) × 5.4 mm (w) × 2.3 mm (t)	16 mm (l) × 4.0 mm (w) × 1.2 mm (t)
Sintering behavior	φ10.0 mm × 5.0 mm (tested on pre-sintered specimens)	

**Table 3:** Composition of Ga solution.

Ga Solution	Composition (wt%)			Ga Content (wt%)
	Distilled Water *	Ga (NO_3_)_3_ · nH_2_O *	Urea *	
GS1.5	65.7	8.5	25.8	1.5
GS2.5	42.7	14.2	43.1	2.5
GS5.0	37.2	28.3	34.5	5.0

* manufactured by FUJIFILM Wako Pure Chemical Corporation, Osaka, Japan.

#### 2.1.1. Preparation of Pre-Sintered Test Piece

Commercially available 5Y zirconia powder containing 0.05 wt% of alumina (Zpex Smile, Tosoh, Tokyo, Japan) was used for a starting powder. The powder was pressed uniaxially by a press molding machine (Sansho Industry, Osaka, Japan) to obtain zirconia blocks having a diameter of 19 mm × thickness of 19 mm and discs having a diameter of 100 mm × thickness of 17 mm. Next, the blocks and discs were isostatically pressed at 200 MPa using a cold hydrostatic isotropic press (Sansho Industry, Osaka, Japan).

The pressed zirconia blocks/discs were degreased at 500 °C in an inert gas oven (INH-21CD, KOYO THERMO, Nara, Japan) and then fired in a firing furnace (SC-3035F, MOTOYAMA, Osaka, Japan) at 970 °C to obtain a pre-sintered zirconia block/disk (φ18 mm × 18 mm/φ98.5 mm × 16 mm).

The pre-sintered zirconia blocks and disks were cut with cutting equipment (Secotom, Struers, Ballerup, Denmark), and surface polished with SiC water resistant polishing paper (# 1000) to reach the dimension required by each test.

#### 2.1.2. Ga_2_O_3_ Doping

The Ga_2_O_3_ doping of each specimen was performed by impregnation with a Ga solution. Table 3 shows the composition of the Ga solution. Individual specimens were immersed in the Ga solution for 8 h to impregnate the Ga solution. After extraction from the solution, they were placed in a vacuum sealer bag and vacuum-packed. The vacuum-packed specimens were heat treated in a convection oven at 97 °C for 15 h. In this process, urea in the Ga aqueous solution is hydrolyzed to ammonia and carbon dioxide, and the pH rises, thereby precipitating gallium ions as gallium compounds. After the heat treatment, each specimen and 4 mL of distilled water per gram of the specimen were placed in an autoclave (HU-100, Sanai Kagaku, Aichi, Japan) and heat-treated at 170 °C for 5 h. This heat treatment step was performed twice for each sample. The specimen was fired at 500 °C for 30 min in a burn out furnace (009-Plus, DENKEN-HIGHDENTAL Co., Ltd., Kyoto, Japan). The gallium nitrate contained in the Ga solution finally changes to Ga_2_O_3_ through the urea hydrolysis process and a firing process at 500 °C.

#### 2.1.3. Sintering of Test Piece

**Figure 2:**
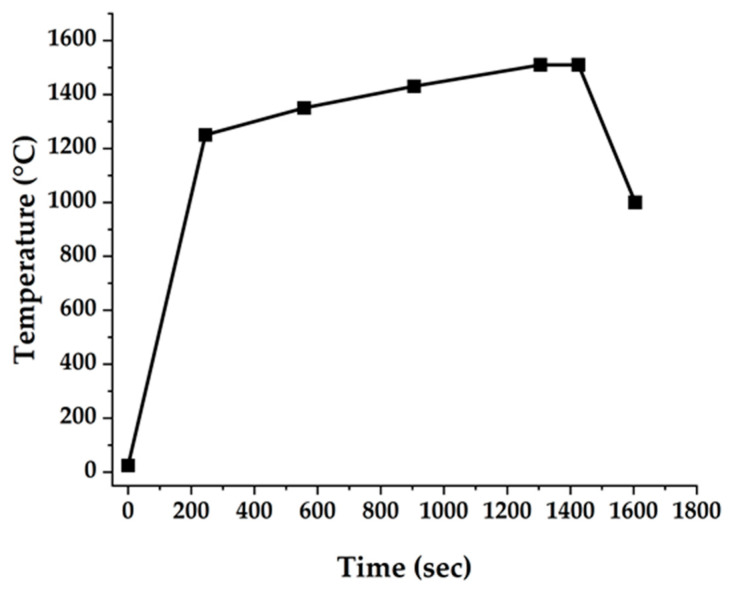
Sintering schedule.

Samples produced as described above were finally subjected to high-speed sintering at 1510 °C according to the sintering schedule shown in Figure 2 using a CEREC Speedfire (Dentsply Sirona, Charlotte, NC, USA).

#### 2.1.4. Processing of Sintered Body

Each sintered specimen was processed to the dimensions required for each test (Figure 1 and Table 2) by a surface grinder (GRIND—X PFG500II, OKAMOTO, Gunma, Japan) using diamond wheels (# 325, # 800). Specimens for XRD measurement and SEM observation were surface-polished with a dental zirconia abrasive ZirGloss (SHOFU INC., Kyoto, Japan). The polished specimens were then heat treated at 1300 °C for 5 min to eliminate the effects of phase transitions and residual stresses due to mechanical polishing. Specimens for the three-point flexural test were surface polished with water-resistant SiC polishing paper (# 1000).

### 2.2. Ga_2_O_3_ Content Evaluation

The Ga_2_O_3_ content in each sample was evaluated by Wavelength dispersion X-ray fluorescence spectroscopy (WDXRF; ZSX100e, Rigaku, Osaka, Japan). The Ga_2_O_3_ concentration was semi-quantified by the fundamental parameter method (FP method) from the fluorescence X-ray data obtained by measuring the surface of each specimen (measuring radius; 10 mm).

### 2.3. Sintering Behavior

The sintering behavior of each sample was evaluated by measuring the shrinkage during sintering with a thermal dilatometer (DIL402 Expedis Classic, NETZSCH, Selb, Germany). The measurement was performed using the temperature range: room temperature to 1600 °C, heating rate: 5 °C/min, atmosphere: air.

### 2.4. Microstructural Characterization

The microstructure of the specimen was evaluated by means of scanning electron microscopy (SEM; JSM6390LA, JEOL, Tokyo, Japan). An acceleration voltage of 15 kV was used. The specimens were Au-coated to prevent charge-up.

The particle size distribution and median diameter (D_50_) of each sample were determined by the image analysis of SEM images. Two hundred grains were randomly selected from the SEM images of each specimen and analyzed using image analysis software (Mac-View, Mountech Co., Ltd., Tokyo, Japan).

Pore density was calculated by counting the number of pores from SEM images in three fields of view of each specimen. Averages were then calculated and converted into values for the unit area.

### 2.5. Density Measurement

The density of each specimen was measured by the Archimedes method (n = 2) according to JIS R 1634: 1998. Relative density was calculated by dividing the measured density by the theoretical density (Equation (1)).

$$Relative\;density=\frac{\rho }{\rho_{0}}\times \;100\;,$$

(1) $$\rho_{0}=\frac{100}{\begin{array}{c} \left(\frac{A}{\rho_{A}}+\frac{G}{\rho_{G}}+\frac{100-A-G}{\rho_{x}}\right) \end{array}}\;,$$

*A*: content of alumina (wt%)
*G*: content of Ga_2_O_3_ (wt%)
*ρ*: measured density (g/cm^3^)
*ρ*_0_: theoretical density (g/cm^3^)
*ρ*_A_: theoretical density of alumina
*ρ*_G_: theoretical density of Ga_2_O_3_
*ρ*_x_: theoretical density of zirconia containing 5.33 mol% of yttria (6.028 g/cm^3^ [21]).

### 2.6. Crystallographic Characterization

The crystal structure of each specimen was characterized by X-ray diffraction (XRD; Multi Flex, Rigaku, Tokyo, Japan) using Cukα radiation as the X-ray source. The specimens used for XRD measurement were the same as those used for XRF measurement. The measurement conditions are as follows: tube current; 40 mA, tube voltage; 40 kV, 2θ range; 20–120 °, scan step; 0.02 °. Rietveld refinement was carried out from the obtained diffraction pattern using a Rietan-FP [22]. Two tetragonal phases with different yttria concentrations were used as the initial structural model for the Rietveld refinement (Table 4) as described in a previous report [23].

The tetragonality (*c/a*) was calculated using the lattice constant refined by Rietveld refinement.

### 2.7. Translucency

The translucency was evaluated by calculating TP (Translucency Parameter) from chromaticity data measured on a white and black background (Equation (2)):

(2) $${TP=[(L}^{*}{}_{w}-L^{*}{}_{b})+(a^{*}{}_{w}-a^{*}{}_{b})+(b^{*}{}_{w}-b^{*}{}_{b}{)\;]}^{1/2},$$

L*, a*, b*: CIE L* a* b* value

Subscript w: measured on the white background
Subscript b: measured on the black background.

The chromaticity value (L* a* b*) of each specimen was measured by a spectroscopic colorimeter (CM-5, Konica Minolta, Hannover, Germany). The specimens were measured on a black and white background (n = 2).

### 2.8. Flexural Strength

The flexural strength was measured by a three-point flexural test in accordance with ISO 6872 (Dentistry-Ceramic materials) (n = 10). The test was performed using a universal testing machine (Instron 5967, INSTRON, Norwood, MA, USA). The test conditions are as follows: span, 12 mm; crosshead speed; 1.0 mm/min. Three-point flexural strength *σ* (MPa) was calculated according to the following equation in accordance with ISO 6872:

(3) $$\sigma =\frac{3Pl}{{2wb}^{2}}\;,$$

*P*: breaking load (N)
*l*: test span (mm)
*w*: width of the specimen (mm)
*b*: thickness of the specimen (mm).

### 2.9. Statistical Analysis

A Tukey-Kramer multiple comparison test was used to compare each sample with regard to relative density, TP and flexural strength.

## 3. Results and Discussion

### 3.1. Ga_2_O_3_ Content

Table 5 shows the Ga_2_O_3_ content of each sample. The Ga_2_O_3_ content in the samples increased with increasing Ga concentration in solution (cf. also Table 3).

### 3.2. Sintering Behavior

**Figure 3:**
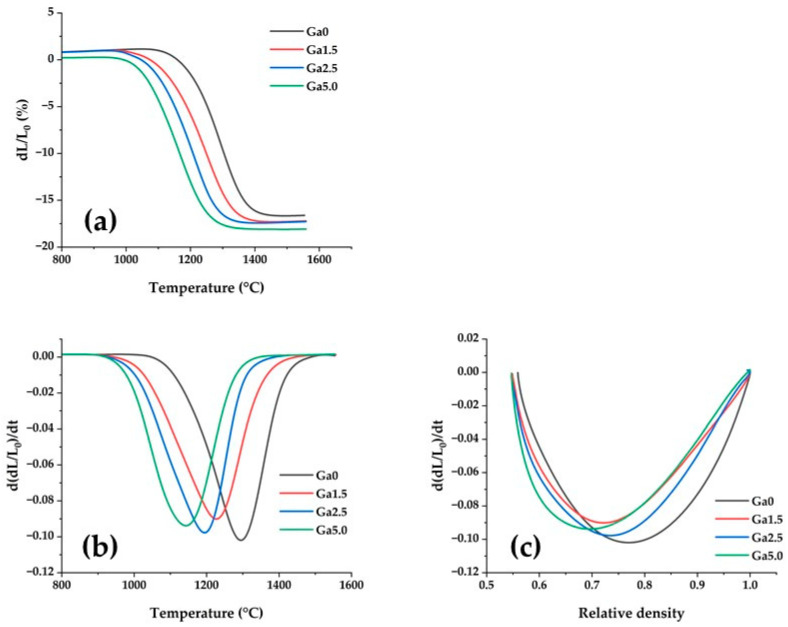
Relationship between: (**a**) shrinkage rate and temperature, (**b**) first derivative of shrinkage rate and temperature, (**c**) first derivative of shrinkage rate and relative density.

The shrinkage curve of each sample is shown in Figure 3a. The shrinkage beginning and ending temperatures decreased with the addition of Ga_2_O_3_. This indicates that Ga_2_O_3_ promotes sintering. Figure 3b shows the first derivative of the shrinkage curve with respect to temperature. The peak height of the first derivative curve for Ga_2_O_3_-added samples was lower than that of the Ga0 one. This indicates that the addition of gallium reduces the maximum sintering rate. Figure 3c shows the first derivative of the shrinkage curve against relative density. The intersection point between the curve for Ga_2_O_3_-added samples and that for Ga0 was at an approximate relative density of 0.7. This result indicates that Ga_2_O_3_-added samples shrink faster than Ga0 at relative densities below 0.7, and slow down after that. In other words, in Ga_2_O_3_-added samples, densification progresses faster than Ga0 from the early stage through the middle stage of sintering, and more slowly than Ga0 after the middle stage of sintering.

Matsui et al. [26] investigated the relationship between yttria concentration in zirconia and sintering behavior, and reported that an increase in yttria concentration leads to a decrease in the sintering rate at the initial stage of sintering and an increase in the sintering rate at the middle to final stage of sintering. Matsui et al. [27] also investigated the effect of alumina addition on the sintering behavior of 3Y zirconia and showed that the addition of alumina increases the maximum sintering rate.

The results of this study indicate that the addition of Ga_2_O_3_ has a different effect on the sintering behavior of yttria-stabilized zirconia than that of yttria or alumina. It is believed that this characteristic effect of Ga_2_O_3_ addition on sintering behavior affects both the formation of residual pores and densification, as discussed in the following section.

### 3.3. Microstructural Characterization

**Figure 4:**
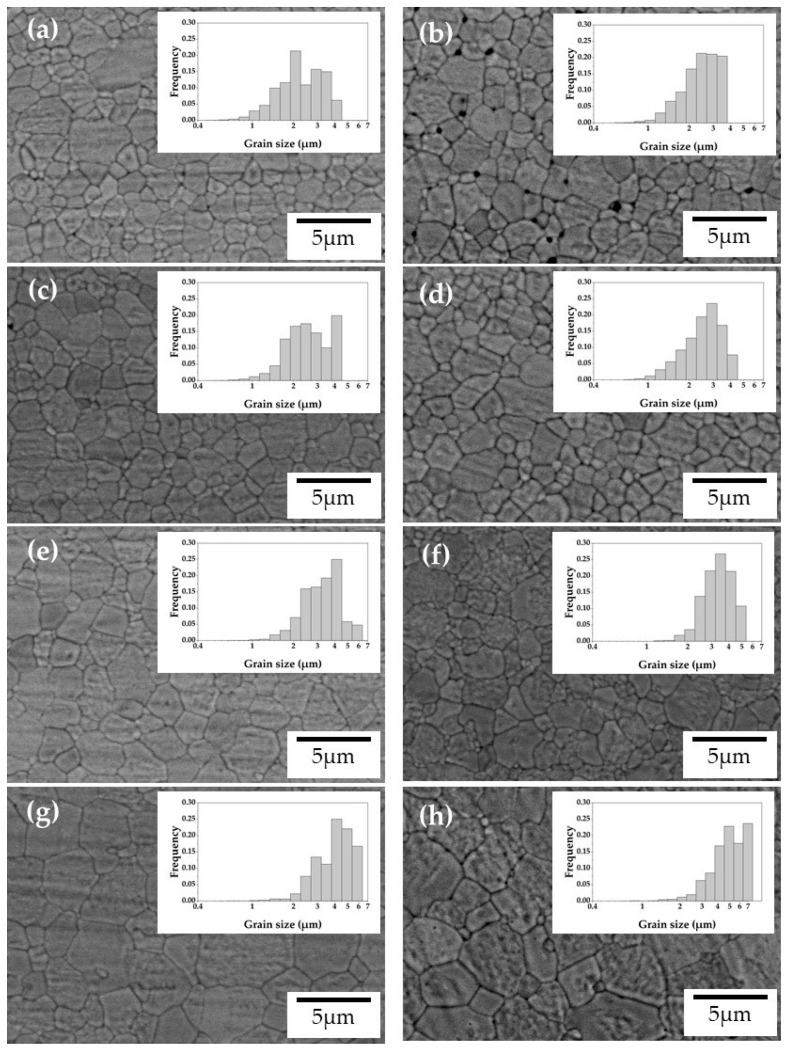
Scanning electron micrographs of: (**a**,**b**) Ga0–1.9 mm, 18 mm, (**c,d**) Ga1.5–1.9 mm, 18 mm, (**e**,**f**) Ga2.5–1.9 mm, 18 mm, (**g**,**h**) Ga5.0–1.9 mm, 18 mm.

**Figure 5:**
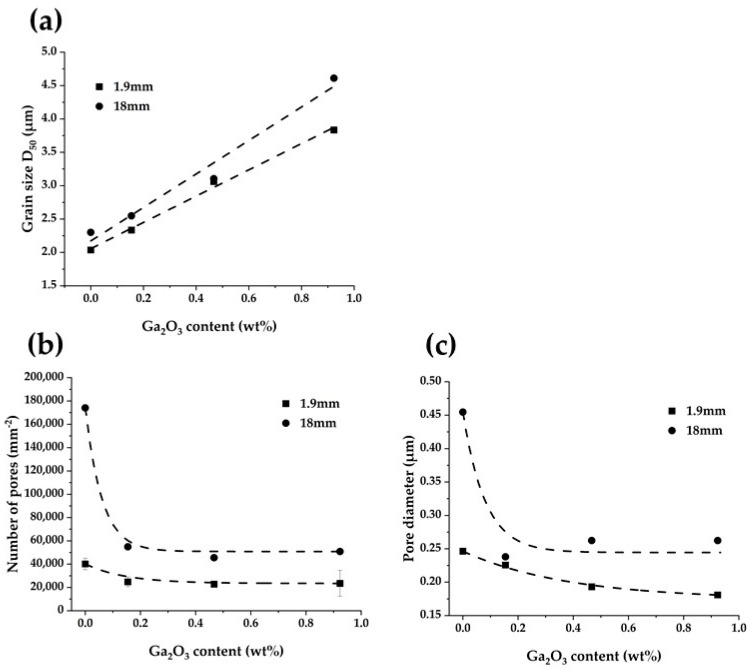
Relationship between (**a**) grain size, (**b**) number of pores, (**c**) pore diameter, and the Ga_2_O_3_ concentration.

Figure 4 shows the SEM images of the investigated samples with pre-sintered thicknesses of 1.9 mm and 18 mm, while Figure 5 shows the relationships of grain size, number of pores, and pore diameter on Ga_2_O_3_ concentration. In the 1.9 mm sample, the number and diameter of pores were the largest at Ga0 and decreased with the increasing Ga_2_O_3_ amount in the sample (Figure 5b,c). In a previous study [15], we reported that high-speed sintered 5Y zirconia had more pores than the conventional sintered one, which reduced the translucency and mechanical properties. The pore number of the Ga_2_O_3_-doped samples in this study was at the same level as that of the conventionally sintered 5Y zirconia in that previous study. Therefore, we can infer here that the addition of Ga_2_O_3_ promotes densification in high-speed sintering. This is due to the change in sintering behavior of 5Y zirconia with the addition of Ga_2_O_3_. Previous studies have suggested that the effects of high-speed sintering on the formation of pores and properties of yttria-stabilized zirconia are greater with higher yttria concentrations [11,12,13,14,15,16]. As mentioned in Section 3.2, as yttria concentration increases, the sintering rate at the early stage of sintering decreases, while the sintering rate increases beyond the middle stage of sintering. Accordingly, one could conclude that the formation of pores in high-speed sintered 5Y zirconia is due to the fact that the addition of yttria causes the rapid densification after the middle stage of sintering, making it difficult for pores to disappear. The addition of Ga has an opposite effect on the sintering behavior of 5Y zirconia compared to the addition of yttria: it slows down the densification rate after the middle stage of sintering, thereby suppressing the residual pores in high-speed sintering.

In Ga0, the number and diameter of pores increased significantly with the increasing thickness of the pre-sintered specimen, while those in the Ga_2_O_3_-added specimen increased only slightly. This indicates that the addition of Ga_2_O_3_ promotes densification in the depth of thick specimens.

The grain size increased as the amount of Ga_2_O_3_ added increased. Matsui et al. [28,29] reported that grain growth in yttria-stabilized zirconia is controlled by the solute drag effect of yttrium ions segregated at grain boundaries. Therefore, the greater the segregation of solute ions to the grain boundaries, the more the grain growth is suppressed. Previous studies [30,31] suggested that cations with low valence segregate at the grain boundaries of zirconia. According to these studies, Ga^3+^ is likely to segregate at grain boundaries during sintering. Therefore, according to the solute drag theory, grain growth should be suppressed by the addition of Ga_2_O_3_. The result, whereby the grain size increased with the addition of Ga_2_O_3_, suggests that the grain growth behavior in this system cannot be explained by the solute drag theory. Yoshida et al. [19] and Matsui et al. [32] reported that the addition of small cations promotes the grain growth of zirconia. This is because small cations segregated at the grain boundaries promote grain boundary diffusion. Ga^3+^ has an ion size of 0.62 Å (6-fold), which is very small compared to Y^3+^ (0.90 Å, 6-fold). Therefore, the increase in grain size by the addition of Ga_2_O_3_ is due to the enhancement of grain boundary diffusion due to the segregated Ga^3+^ at the grain boundaries. As the amount of Ga_2_O_3_ added increases, the amount of Ga^3+^ segregated to the grain boundaries also increases, and the grain boundary diffusion enhancement effect becomes greater. Therefore, the grain size increased as the amount of Ga_2_O_3_ added increased.

In all samples, the grain size increased as the thickness of the pre-sintered specimen increased (Figure 5a). This is because the temperature inside the specimen is higher than that on the surface. A Speedfire microwave heating furnace was used in this study. Therefore, the specimen that absorbs the microwaves heats up during sintering. On the other hand, there is no heat source around the specimen, and thus the temperature of the specimen surface is lower than that inside the specimen. This phenomenon is known as “inverse temperature gradient” [33]. As a result, the grain size at the center of the specimen is larger due to greater ion diffusion inside the specimen than at the surface [34].

### 3.4. Density

**Figure 6:**
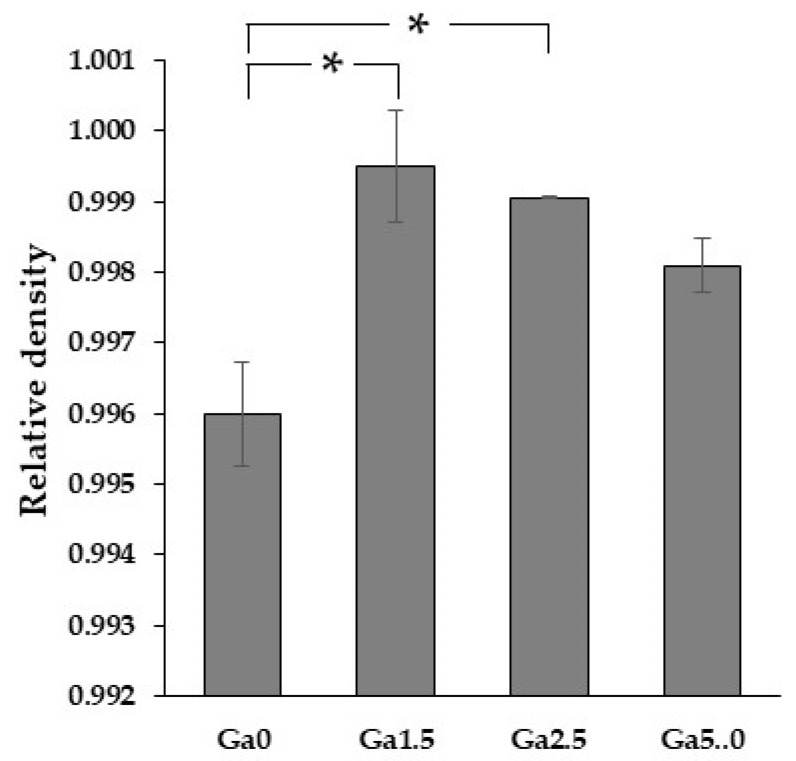
Relative densities of each sample. The asterisk refers to statistical significance according to Tukey-Kramer multiple comparison test.

Figure 6 shows the relationship between Ga_2_O_3_ content and relative density for a fixed sintering schedule. Ga_2_O_3_-doped samples showed higher relative densities than Ga0. This indicates that Ga_2_O_3_ doping promoted densification. The relative density was highest for Ga1.5 and decreased slightly with increasing Ga_2_O_3_ content. However, as described in Section 3.3, SEM observations did not confirm that Ga5.0 has more pores than Ga1.5 and Ga2.5. This decrease in relative density, accompanied by an increase in Ga_2_O_3_ content, is likely related to the calculation method of theoretical density. In this study, the theoretical density was calculated by Equation (1). This formula provides the density of a mixture of alumina, Ga_2_O_3_ and 5Y zirconia. However, the results of an X-ray diffraction analysis (Section 3.5) suggest that Ga_2_O_3_ is dissolved in 5Y zirconia. When Ga_2_O_3_ is dissolved in 5Y zirconia, Ga_2_O_3_ has the same crystal structure as 5Y zirconia, which is different from the original Ga_2_O_3_ crystal structure. Furthermore, when Ga^3+^ replaces Zr^4+^, oxygen vacancies are generated for charge compensation. Therefore, the theoretical density calculated for the mixture may differ slightly from the theoretical density of the actual specimen. Ingel et al. [21] reported that in yttria-stabilized zirconia, the theoretical density of a mixture of pure zirconia and yttria was higher than that of the actual yttria-stabilized zirconia. If it is the same when Ga_2_O_3_ is dissolved, the theoretical density of Ga_2_O_3_-doped 5Y zirconia obtained by Equation (1) may be calculated to be larger than the actual theoretical density, and this difference is expected to increase with increasing Ga_2_O_3_ concentration. We believe that this is the reason for the decrease in relative density in the Ga1.5-Ga5.0 range.

### 3.5. Crystallographic Characterization

**Figure 7:**
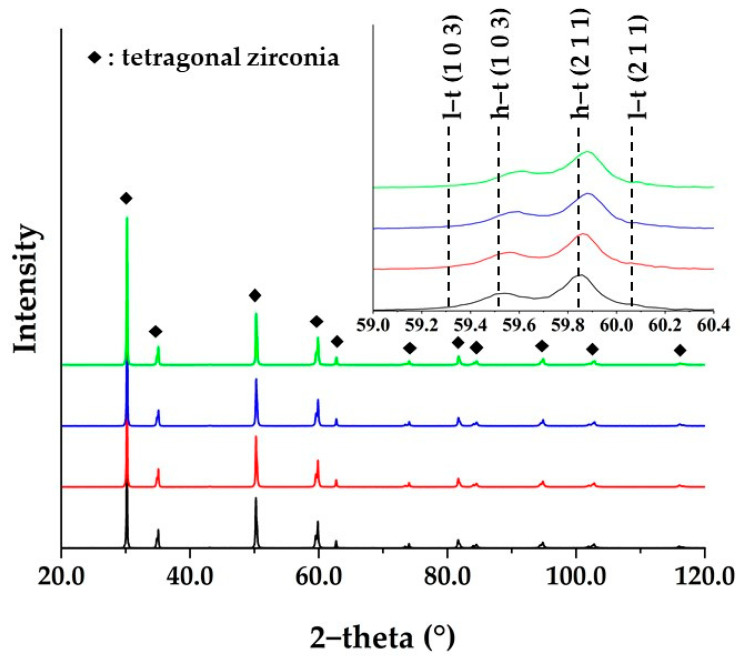
X-ray diffraction patterns of each sample. Diamonds indicate the diffraction peaks of the high and/or low yttrium tetragonal phase of yttria stabilized zirconia. The inset shows an enlarged view from 59.0° to 60.4°. Black line; Ga0, red line; Ga1.5, blue line; Ga2.5, green line; Ga5.0. The h-t and l-t used to indicate the Miller index in the inset indicate high yttrium tetragonal and low yttrium tetragonal, respectively.

**Figure 8:**
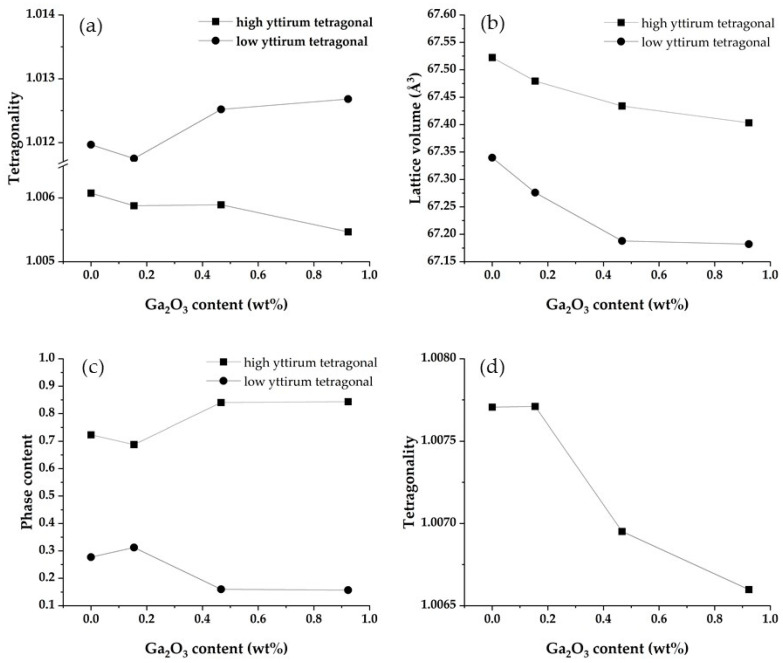
Relationship between (**a**); tetragonality, (**b**); lattice volume, (**c**); phase contents, and (**d**); mean tetragonality as calculated by Equation (4) and Ga_2_O_3_ concentration.

Figure 7 shows the X-ray diffraction pattern of the investigated samples (cf. sample description in caption). No significant difference in X-ray diffraction intensity was observed between these samples. This indicates that the addition of Ga_2_O_3_ does not affect the crystallinity of the zirconia sintered bodies. The X-ray diffraction peaks of all samples were assigned to only two tetragonal phases: a high yttrium tetragonal phase and a low yttrium tetragonal phase. No diffraction peaks from Ga compounds (e.g., Ga_2_O_3_) were detected. An additional feature was that the diffraction peak of high yttrium tetragonal shifted towards higher angles upon the addition of Ga_2_O_3_. This suggests a decrease in lattice volume of tetragonal zirconia with the addition of Ga_2_O_3_. Accordingly, it is believed that the added Ga_2_O_3_ is dissolved in the zirconia crystal lattice. Table 6 shows the weight ratio of the two tetragonal phases, their tetragonality, and the lattice volume, and Figure 8 shows the relationship between them and the Ga_2_O_3_ content.

The tetragonality of high yttrium and low yttrium tetragonal phases displayed a contrasting behavior, with the tetragonality of the high yttrium tetragonal phase decreasing with increasing Ga_2_O_3_ content, while showing the opposite trend for the low yttrium tetragonal phase. In addition, the content of the high yttrium tetragonal phase increased with the addition of Ga_2_O_3_. These results suggest that phase separation into a high yttrium tetragonal and low yttrium tetragonal phase may be promoted with the addition of Ga_2_O_3_. It is known that yttria-stabilized zirconia begins to separate into yttria-rich and poor regions above 1300 °C, which eventually become high yttrium and low yttrium tetragonal phases [24,28,29]. Although the mechanism by which the addition of Ga_2_O_3_ promotes phase separation is not clear, it is expected to be due to the enhancement of grain boundary diffusion due to the segregation of Ga^3+^ to the grain boundaries. As discussed in Section 3.3, Ga^3+^ is expected to segregate at the grain boundaries. In addition, small cations segregated at the grain boundaries of zirconia improve grain boundary diffusion [19,32]. Therefore, the Ga^3+^ segregated at the grain boundaries promoted the diffusion of Y^3+^, resulting in enhanced phase separation.

As mentioned above, the tetragonality of the two tetragonal phases showed an opposing behavior, so the stabilization behavior of the system as a whole is unclear. Therefore, we calculated the mean tetragonality as the tetragonality of the entire system using Equation (4).

(4) $$mean\;tetragonality=t_{h}\;\times \;c_{h}+t_{l}\times c_{l}\;,$$

*t*: tetragonality
*c*: phase content
Subscript *h*: high yttrium tetragonal phase
Subscript *l*: low yttrium tetragonal phase

The mean tetragonality decreased with increasing Ga_2_O_3_ content (Figure 8d). This indicates that the whole system was stabilized with increasing Ga_2_O_3_ concentration. That is, as the Ga_2_O_3_ concentration increased, the low yttrium tetragonal phase became unstable, but the high yttrium tetragonal phase became stable, and the content of that increased, and therefore the entire system was stabilized. It is therefore concluded that Ga_2_O_3_ functions as a stabilizer, which is consistent with the report by Li et al. [35].

Lattice volume decreased with increasing Ga_2_O_3_ content in both high yttrium and low yttrium tetragonal phases. There are two possible reasons for the decrease in lattice volume: the decrease in yttrium concentration [21], and the solid solution of Ga_2_O_3_. The tetragonality increases as the yttrium concentration decreases. In the high yttrium tetragonal phase, the tetragonality decreased with the addition of Ga_2_O_3_, indicating that the yttria concentration did not decrease. Therefore, the lattice volume reduction in the high yttrium tetragonal phase is due to the solid solution of Ga_2_O_3_. When Ga_2_O_3_ forms a solid solution in 5Y zirconia, there are two possible behaviors of Ga^3+^: Ga^3+^ replaces Zr^4+^ or Y^3+^. Here, Ga^3+^ has a smaller ionic radius than Zr^4+^ and therefore has less ability as a stabilizer than Y^3+^ [35]. Thus, if Ga^3+^ replaces Y^3+^, the tetragonality is expected to increase (i.e., destabilize). Since the tetragonality was reduced in the high yttrium tetragonal phase, it is considered that Ga^3+^ replaced Zr^4+^.

On the other hand, in the low yttrium tetragonal phase, the tetragonality increased, indicating a decrease in yttrium concentration. Therefore, the decrease in lattice volume in the low yttrium tetragonal phase could be due to either the decrease in yttrium concentration or the solid solution of Ga_2_O_3_, or both, which cannot be distinguished in the results of this study.

The crystallographic effects of the addition of Ga_2_O_3_ are summarized below.

- Ga_2_O_3_ dissolves in 5Y zirconia and reduces the lattice volume;
- Dissolved Ga_2_O_3_ functions as a stabilizer;
- Ga_2_O_3_ promotes the phase separation into a high yttrium tetragonal and low yttrium tetragonal phase.

A more detailed crystallographic investigation is necessary to clarify the relationship between the gallium addition and the crystalline phase composition of zirconia.

### 3.6. Translucency

**Figure 9:**
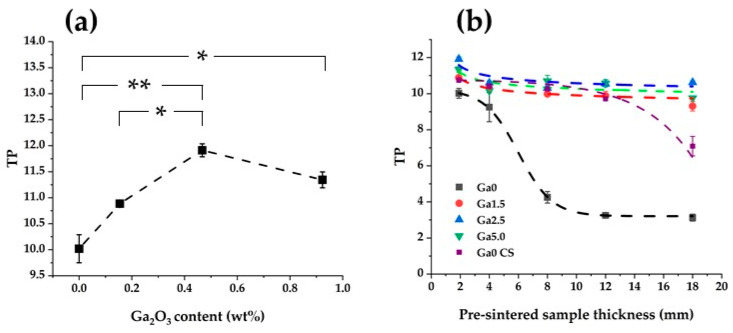
Relationship between TP of each sample and (**a**) Ga_2_O_3_ concentration, (**b**) Pre-sintered sample thickness. The asterisk and two asterisks in (**a**) refer to statistical significance. Ga0 CS in (**b**) indicates conventional sintered Ga0, the data of which is taken from previous study [15].

Figure 9a shows the relationship between the translucency and the Ga_2_O_3_ concentration of a sample with a pre-sintered thickness of 1.9 mm. This result proves that the addition of Ga_2_O_3_ improved the translucency. This effect is considered to be a consequence of both microstructural change and crystal phase composition. As mentioned above, the addition of Ga_2_O_3_ decreased both the number and diameter of residual pores. The translucency of zirconia is sensitive to pore size and concentration, and even a small pore reduces translucency [36]. Therefore, the addition of Ga_2_O_3_, which reduced both the number and diameter of pores, improved translucency. In addition, a decrease in grain boundary scattering due to the larger grain size might also contribute to the observed improvement in translucency.

Regarding crystal phase composition, the decrease in tetragonality and the increase in the proportion of high yttrium tetragonal phase due to the addition of Ga_2_O_3_ led to the improvement of translucency. In general, ceramic polycrystals have higher translucency, as the optical anisotropy of the crystal is smaller. XRD results show that all samples consist of optically anisotropic tetragonal phases (Figure 7, Table 6). A tetragonal crystal becomes closer to an optically isotropic cubic phase as the tetragonality decreases. As a result, light scattering due to optical anisotropy is reduced and translucency is improved. From the above, the addition of Ga_2_O_3_ led to a decrease in tetragonality and an increase in the ratio of high yttrium tetragonal phases close to the cubic phase, which resulted in the suppression of light scattering and an improvement in translucency.

Figure 9b shows the relationship between translucency and specimen pre-sintered thickness. In the case of Ga0, the translucency decreased significantly with increasing specimen pre-sintered thickness. In addition, in previous studies [15], even conventional sintered 5Y zirconia showed a decrease in translucency at a pre-sintered specimen thickness of 18 mm. On the other hand, there was almost no decrease in the Ga_2_O_3_-added sample. As mentioned above, the SEM observation revealed that the diameter and number of pores increased significantly with the increasing pre-sintered thickness of the specimen in the case of Ga0. This increase in the diameter and number of pores significantly reduced the sample translucency. On the other hand, in Ga_2_O_3_-added samples, the increase in the diameter and number of pores with increasing specimen pre-sintered thickness was small, thus almost no decrease in translucency was observed.

In clinical practice, the thickness of a dental prosthesis may exceed 10 mm depending on the case, so it is important to be able to maintain translucency even in thick specimens. The Ga_2_O_3_-added sample maintained translucency better than Ga0 and even better than that of conventional sintered 5Y zirconia. Ga_2_O_3_-doping is thus a promising approach for dental zirconia.

### 3.7. Flexural Strength

**Figure 10:**
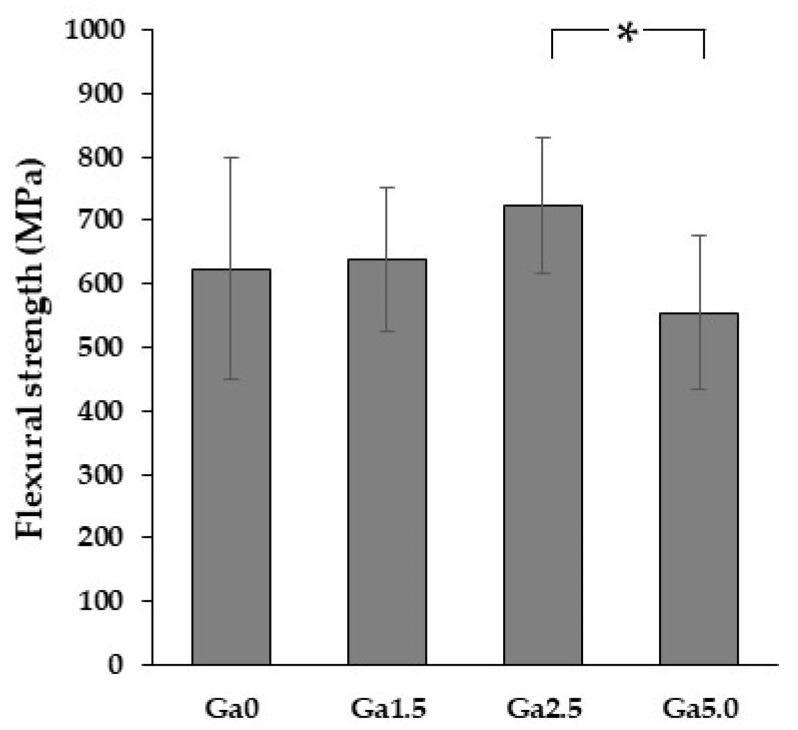
Flexural strength of each sample. The asterisk refers to statistical significance.

Figure 10 shows the three-point flexural strength of each specimen. The flexural strength slightly increased with the addition of Ga_2_O_3_ and showed a maximum value at Ga2.5. Similar to the translucency trend described above, the change in flexural strength is also caused by both the microstructure and crystalline phase composition. In polycrystalline ceramics, defects such as residual pores act as fracture initiation points, so the size and number of residual pores have a large effect on flexural strength. Density measurements and SEM observations show that the size and number of residual pores decreased upon adding Ga_2_O_3_. This is the reason for the observed improvement in flexural strength associated with Ga_2_O_3_-doping. On the other hand, an increase in particle size due to the addition of Ga_2_O_3_ reduces flexural strength [37].

From a crystallographic viewpoint, the observed change in crystal phase composition upon the addition of Ga_2_O_3_ is thought to have a negative effect on flexural strength. The high flexural strength and fracture toughness of yttria partially stabilized zirconia polycrystals are attributed to tetragonal→monoclinic stress-induced phase transformation; accordingly, the higher the content of low yttrium tetragonal phase with high tetragonality (i.e., the phase at the origin of stress-induced phase transformation), the higher flexural strength and fracture toughness [23]. XRD results showed that the addition of Ga_2_O_3_ decreased the tetragonality and the content of the low yttrium tetragonal phase. This, in turn, makes the stress-induced phase transformation less likely and lowers the flexural strength.

In summary, Ga_2_O_3_ addition affects the flexural strength in both a positive way by decreasing the number and size of pores, and in a negative way by increasing grain size and decreasing the amount of stress-induced phase transition. In this study, Ga2.5 showed the highest value flexural strength because of the balance of these two contrasting effects.

The flexural strength of the Ga_2_O_3_-added samples was 555~724 MPa. This strength level complies with ISO6872 Class 4 (>500 MPa; usable for connected prostheses with three teeth). Since ‘One Visit Treatment’ does not treat cases larger than 3-unit-prostheses, Ga_2_O_3_-added samples have clinically suitable flexural strength. Furthermore, the Ga_2_O_3_-added samples show less of an increase in pore number and diameter, even in thick specimens, and high reliability is obtained, even in thick prostheses. Therefore, Ga_2_O_3_-added 5Y zirconia is a promising material for high-speed sintering.

## 4. Conclusions

This study revealed that the addition of Ga_2_O_3_ to 5Y zirconia improves translucency and flexural strength during high-speed sintering. The crystallographic characterization by XRD indicated that the added Ga_2_O_3_ dissolved in 5Y zirconia, leading to a decrease in tetragonality and an increase in the content of high yttrium tetragonal phases. Microstructural evaluation by SEM showed that the addition of Ga_2_O_3_ reduced the amount and size of pores in the sintered body, especially at the center of thick specimens. It was concluded that changes in the composition of these crystal phases and microstructure led to appreciable improvements in translucency and flexural strength.

The measurement of the shrinkage rate with a dilatometer showed that the addition of Ga_2_O_3_ increased the sintering rate from the early to middle stages of sintering, and decreased it beyond the middle stage. It was concluded that this change in sintering behavior was key to reducing both the number and size of pores in the sintered body.

Ga_2_O_3_-added 5Y zirconia exhibits high translucency and flexural strength, even when a thick specimen is sintered at high speed, making it a suitable material for one-visit treatment.

## Figures and Tables

**Table 4:** ICDD cards used for refinement of the specimen.

Crystal Phase	Chemical Formula	Space Group	ICDD Number	Authors
4Y tetragonal (low yttrium region)	(ZrO_2_)_0.96_(Y_2_O_3_)_0.04_	P4_2_/nmc	00-060-0503	Yamashita et al. [24]
5Y tetragonal	(ZrO_2_)_0.95_(Y_2_O_3_)_0.05_	P4_2_/nmc	01-070-4428	Lamas and Walsöe De Reca. [25]

**Table 5:** Ga_2_O_3_ content of each sample.

Sample Name	Ga_2_O_3_ Content (wt%)
Ga0	0.000
Ga1.5	0.154
Ga2.5	0.467
Ga5.0	0.923

**Table 6:** Tetragonality, lattice volume and phase contents of each sample.

		Ga0	Ga1.5	Ga2.5	Ga5.0
tetragonality	high yttrium tetragonal	1.0061	1.0059	1.0059	1.0055
	low yttrium tetragonal	1.0120	1.0117	1.0125	1.0127
lattice volume (Å^3^)	high yttrium tetragonal	67.52	67.48	67.43	67.40
	low yttrium tetragonal	67.34	67.28	67.19	67.18
phase content (wt%)	high yttrium tetragonal	0.72	0.69	0.84	0.84
	low yttrium tetragonal	0.28	0.31	0.16	0.16

## Data Availability

The datasets generated and/or analyzed during the current study are available from the corresponding author on reasonable request.
